# Is pathology necessary to predict mortality among men with prostate-cancer?

**DOI:** 10.1186/s12911-014-0114-6

**Published:** 2014-12-12

**Authors:** David Margel, David R Urbach, Lorraine L Lipscombe, Chaim M Bell, Girish Kulkarni, Jack Baniel, Neil Fleshner, Peter C Austin

**Affiliations:** Division of Urology, Rabin Medical Center, Beilinson Campus, 39 Jabotinsky, Petah Tikva, 4941492 Israel; Davidoff Cancer Center, Rabin Medical Center, Beilinson Campus, Petah Tikva, Israel; Departments of Surgery and Health Policy Management and Evaluation, University of Toronto, Toronto, Canada; Division of Clinical Decision Making and Health Care, Toronto General Hospital Research Institute, Toronto, Canada; Institute for Clinical Evaluative Sciences (ICES), Toronto, Canada; Cancer Care Ontario, Ontario, Canada; Institute for Health Policy Management and Evaluation, University of Toronto, Toronto, Canada; Department of Medicine, Women’s College Hospital and Research Institute, University of Toronto, Toronto, Canada; Department of Medicine and Keenan Research Centre in the Li Ka Shing Knowledge Institute, St. Michael’s Hospital, Toronto, Canada; Division of Urology, Department of Surgical Oncology, Princess Margaret Hospital, University Health Network, Toronto, Ontario Canada

**Keywords:** Prostate cancer, Survival, Prediction models, Population-based study

## Abstract

**Background:**

Statistical models developed using administrative databases are powerful and inexpensive tools for predicting survival. Conversely, data abstraction from chart review is time-consuming and costly. Our aim was to determine the incremental value of pathological data obtained from chart abstraction in addition to information acquired from administrative databases in predicting all-cause and prostate cancer (PC)-specific mortality.

**Methods:**

We identified a cohort of men with diabetes and PC utilizing population-based data from Ontario. We used the c-statistic and net-reclassification improvement (NRI) to compare two Cox- proportional hazard models to predict all-cause and PC-specific mortality. The first model consisted of covariates from administrative databases: age, co-morbidity, year of cohort entry, socioeconomic status and rural residence. The second model included Gleason grade and cancer volume in addition to all aforementioned variables.

**Results:**

The cohort consisted of 4001 patients. The accuracy of the admin-data only model (c-statistic) to predict 5-year all-cause mortality was 0.7 (95% CI 0.69-0.71). For the extended model (including pathology information) it was 0.74 (95% CI 0.73-0.75). This corresponded to a change in category of predicted probability of survival among 14.8% in the NRI analysis.

The accuracy of the admin-data model to predict 5-year PC specific mortality was 0.76 (95% CI 0.74-0.78). The accuracy of the extended model was 0.85 (95% CI 0.83-0.87). Corresponding to a 28% change in the NRI analysis.

**Conclusions:**

Pathology chart abstraction, improved the accuracy in predicting all-cause and PC-specific mortality. The benefit is smaller for all-cause mortality, and larger for PC-specific mortality.

## Background

Administrative databases are often used to create models to predict clinical outcomes, in particular survival [1-4]. Most cancers are fast growing, and once diagnosed have an enormous impact on survival. Therefore, commonly, models to predict survival among these subjects include detailed oncologic information [5-7]. However, earlier cancer diagnoses and advances in treatment have been associated with reduced cancer mortality, such that in 2003 there were an estimated 10 million cancer survivors in the United States [8]. Consequently, patients are living longer after a diagnosis of cancer to the point where existing comorbidities may have a substantial impact on their overall survival.

Prostate cancer is the most common form of non skin cancer diagnosed in men, with three quarters of cases occurring in men aged 65 years and older [9,10]. Prostate cancer is slow growing. Accordingly, death among prostate cancer patients is more likely to be associated with a subject’s comorbidities than prostate cancer itself [11-13]. This is particularly true among patients with diabetes [14,15].

Capturing information from pathology data is labor intensive and expensive. Therefore, if the addition of these pathology clinical variables to a predictive model with variables attained solely from administrative data does not enhance model performance, their inclusion should be avoided. The objective of this study was to quantify the impact of adding Gleason grade and cancer volume (obtained from chart review) to a predictive model for mortality among a cohort of elderly men with incident diabetes and prostate cancer. We further aimed to distinguish all-cause mortality from prostate-cancer-specific mortality. We hypothesized that pathology data may have a great impact on disease-specific mortality, but a smaller or even a null effect on all-cause mortality.

## Methods

### Overview

This cohort was used in a prior study to examine the impact of diabetes on prostate cancer survival [16]. We are therefore able to use it to determine the incremental utility of pathological data, obtained from chart abstraction, in addition to demographic and co-morbidity information, acquired from administrative databases, in predicting all-cause and prostate-cancer-specific mortality among diabetic men with prostate cancer. The current study was approved by the institutional review boards at Sunnybrook and Princess Margret Hospital, University Health Network, Toronto, Canada.

### Data sources

The province of Ontario has a population of approximately 13 million. All residents are covered under a universal health insurance plan. Individuals aged 65 or older are additionally eligible for prescription drug coverage. We used a variety of electronic health data resources linkable using an encrypted unique identifier. The Ontario Cancer Registry (OCR) is a computerized database of Ontario residents newly diagnosed with cancer (except non-melanoma skin cancer), which is estimated to be more than 95% complete [17]. The Ontario Diabetes Database (ODD) - a validated, administrative data-derived registry of diabetes cases in Ontario [18]. The Ontario Health Insurance Plan (OHIP) database includes claims paid to physicians, groups, laboratories, and out-of-province providers [19]. The Canadian Institute for Health Information (CIHI) Discharge Abstract Database (DAD) contains records for each hospital stay [20]. The CIHI National Ambulatory Care Reporting System (NACRS) captures information on ambulatory care, including day surgery, outpatient clinics, and emergency departments. CIHI DAD, NACRS and OHIP were used to capture comorbidities. The Registered Persons Data Base (RPDB) contains demographics and vital status.

### Study population and cohort definition

Our study cohort consisted of men aged 66 years or older with incident diabetes and subsequent PC, identified using the following steps. First, we used the ODD to identify all patients aged 66 years or older with incident diabetes in Ontario between April 1, 1994 and March 31, 2008 (160,867 subjects). We then crossed referenced with OCR to identify patients with incident PC after diabetes diagnosis (ICD-9 code 185, and ICD-10 code C61). We excluded patients diagnosed with PC prior to diabetes and patients with non-prostate malignancies (25,882 subjects). We then performed a detailed chart review of all pathology reports available from OCR and excluded patients without pathology data (855 subjects). Cohort entry date was defined as date of PC diagnosis. We followed up eligible individuals until they experienced an event (PC-specific or all-cause mortality), or among those who did not die, a last health services contact in Ontario (for those who lost health contact for at least 6 months) or March 31, 2009, whichever came first.

### Pathology chart abstraction

For the purpose of this study we performed a pathology chart abstraction of all subjects with available pathology reports at OCR. Chart abstraction was performed by an expert Uro-Oncologist (DM). The variables abstracted were Gleason grade (primary and secondary), volume of tumor (in biopsy the volume was defined as number of positive cores/ number of cores taken; in prostatectomy specimens the volume as recorded by the pathologist), PSA value at biopsy and procedure (i.e. prostate biopsy or transurethral prostatectomy). These variables were linked deterministically to our dataset using the unique OCR identifier. Since only 1312 (28.6%) had a PSA value recorded this variable was not included in the analysis.

For subjects with repeat biopsies during follow-up the first biopsy was considered the “diagnostic biopsy”. Any pathology report from an external consultant was documented and included in the analysis. Subjects who had a transurethral prostatectomy and subsequently a prostate biopsy were considered diagnosed by transurethral resection (TUR) but the Gleason score and tumor volume were assigned based on prostate biopsy.

### Outcome definitions

We measured 2 outcomes: (1). PC-specific mortality, which was derived from the cause of death recorded in the OCR. The cause of death variable in OCR has been validated in several previous studies [21-23]. (2). All-cause mortality was derived from death certificate data in RPDB. The date of death for both outcomes was taken from the RPDB.

### Covariates and statistical analysis

In this study we compared two nested Cox- proportional hazard models to model the hazard of all-cause and prostate cancer-specific mortality. The first model consisted of covariates that were derived from administrative databases including: age at prostate cancer diagnosis, Johns Hopkins Clinical Groups Case Mix system weighted sum of Adjusted Diagnostic Group [24,25], year of cohort entry (to adjust for temporal changes in the management of diabetes and prostate cancer), socioeconomic status (SES) and rural housing. Socioeconomic status and housing status were assessed using the RPDB. The RPDB uses information from Stats Canada and links each individual postal code to an appropriate defined neighborhood. Statistics Canada reports the median income for each neighborhood, and ranks them into quintiles (5 refer to the highest socioeconomic status whereas quintile 1 is the lowest). The second model included Gleason grade and cancer volume (obtained from pathology abstraction) in addition to all aforementioned variables. We used the c-statistic described by Pencina et al. [26-28] as a measure of the accuracy of the two models. We also used net-reclassification improvement (NRI) as a measure of the utility of the added pathology variables. Using the NRI method [26,29], we first used the fitted model that used administrative data only, to classify persons’ predicted probability of 5-year mortality into low (less the 10%), intermediate (10-50%) or high (more than 50%) risk. We chose these categories as we believe they represent clinically meaningful categories; similar categories were used by others to asses all-cause mortality among prostate cancer patients [30]. Subsequently, we repeated the same classification process using the extended model (i.e. with the addition of Gleason grade and cancer volume). If the first model categorized the individual’s predicted risk of 5 year mortality to the intermediate category (10-50%), for example, and the second model categorized the same individual into the high category ( more than 50%), the new model moves the predicted risk score ‘UP’. If, however, the new model categorized the individual into the low risk category, the model moves the predicted risk score ‘DOWN’. We repeated the same analysis for all-cause and prostate-cancer specific mortality. The c-statistic and NRI were calculated using a macro provided Chambless et al. [31] and 95% CI were estimated using 200 bootstrap samples. All analyses were conducted with SAS version 9.2 (SAS Institute).

## Results and discussion

Overall, 4856 incident diabetic men older than 66 who subsequently developed prostate cancer were identified. Pathology reports were available for 4001 (82%) who were included in the analysis. The median (IQR) age at PC diagnosis was 75 (72–79) years (Table 1). During a median (IQR) follow up of 4.7 (2.7-7.3) years, 1395 (35%) individuals died, with 321 patients dying of PC (8.5%). At time of PC diagnosis, 1007 patients (25.2%) had high grade (Gleason score ≥ 8) and 2245 (56%) had high volume tumors (tumor volume ≥ 30%).

**Table 1:** **Baseline cohort (n = 4001)**

**Age (years)at index date, median (IQR)**		75 (72-79)
**Follow-up time (years), mean (SD) before prostate cancer**		2.9 (1.2-5.2)
**Follow-up time (years), mean (SD) from prostate cancer to end of follow up**		4.7 ( 2.7-7.3)
**Gleason grade at presentation**	**Low grade**	1574 (39.3%)
	**Intermediate**	1420 (35.4%)
	**High grade**	1007 (25.2%)
**Primary treatment**	**Surgery**	317 (7.9%)
	**Radiation**	937 (33.2%)
	**Watchful waiting**	1740 (43.5%)
	**ADT**	1329 (46%)
**Volume of prostate cancer**	**High (>30%)**	2245 (56%)
	**Low (≤30%)**	1753 (44%)
**TUR diagnosis n (%)**		681 (18%)
**Co morbidity sum of ADGs n (%)**	**5 or less**	1212 (30.3%)
	**6-9**	1951 (49%)
	**10 or more**	838 (20.7%)
*SES status n (%)	**1**	769 (20.3%)
	**2**	838 (22.2%)
	**3**	764 (20.2%)
	**4**	693 (18.3%)
	**5**	717 (19%)
**Urban n (%)**		545 (85.6%)
**Prostate cancer specific death n (%)**		321 (8.5%)
**Overall mortality n (%)**		1395 (35%)

The multivariable models to predict all-cause mortality are described in Tables 2 and 3 (Table 2- the model consisting of variables derived from administrative data only; Table 3- the model that included Gleason grade and cancer volume in addition to variables derived from administrative data). In both models age, year of cohort entry, rural residence and comorbidities were independent predictors of all-cause mortality. Higher Gleason grade and cancer volume (Table 3) were also associated with increased all-cause mortality, even after controlling for the effect of other variables. The accuracy of the models (i.e. c-statistic) to predict 5-year mortality were 0.7 (95% CI 0.69-0.71) and 0.74 (95% CI 0.73-0.75) for the admin-data only and the extended model (including pathology information), respectively. This corresponded to an incremental increase of 0.04 (95% CI 0.03-0.05) in the c-statistic.

**Table 2:** **Administrative data only model to predict all-cause mortality**

		**HR 95% CI**	**p**
Age		1.113 (1.102-1.123)	<0.0001
Year of cohort entry		0.952 (0.937-0.967)	<0.0001
Rural		1.28 (1.12-1.47)	0.0003
Co morbidity ADGs	Low	Ref	
	Intermediate	1.3 (1.14-1.5)	<0.0001
	High	1.64 (1.45-1.87)	<0.0001
SES status	1	Ref	
	2	0.941 (0.81-1.1)	0.43
	3	0.94 (0.8-1.1)	0.42
	4	0.78 (0.66-0.92)	0.004
	5	0.87 (0.75-1.0)	0.11

Model c statistic to predict 5 year all-cause mortality: 0.7 (95% CI 0.69-0.71).

**Table 3:** **Extended model with pathology to predict all-cause mortality**

		**HR 95% CI**	**p**
Age		1.1 (1.093-1.15)	<0.0001
Year of cohort entry		0.95 (0.93-0.96)	<0.0001
Rural		1.29 (1.13-1.48)	0.0002
Co morbidity ADGs	Low	ref	
	Intermediate	1.31 (1.15-1.51)	<0.0001
	High	1.65 (1.45-1.89)	<0.0001
SES status	1	ref	
	2	0.94 (0.81-1.1)	0.43
	3	0.99 (0.85-1.17)	0.93
	4	0.78 (0.66-0.92)	0.004
	5	0.85 (0.72-0.99)	0.05
Gleason grade	Low	ref	
	Intermediate	1.16 (1.01-1.3)	0.04
	High	2.3 (1.97-2.64)	<0.0001
Volume of prostate cancer	Low (≤30%)	ref	
	High ( >30%)	1.14 (1.01-1.29)	0.036

Model c statistic to predict 5 year all-cause mortality: 0.74 (95% CI 0.730-0.76).

Using the NRI method, we first used the model based on administrative data only, to classify persons’ predicted probability of 5-year mortality into low (less the 10%), intermediate (10-50%) or high (more than 50%) risk. The risk category of predicted probability of 5-year mortality did not change in 85.2% of patient when the extended model was applied (Table 4). Among the 214 (5.3%) subjects with low predicted probability of 5-year mortality, the extended model reclassified 31 (14.5%) subjects to a higher risk group (Table 4). Among the 353 (8.8%) patients with a high predicted probability of 5-year mortality, the extended model moved 122 (34.6%) patients to a lower risk group. Of the 3432 patients classified to 10-50% risk, the extended model moved 221 (6.4%) to the lower risk group and 219 (6.4%) to the higher risk group.

**Table 4:** **Net reclassification improvement**

**Basic model risk levels**	**Extended model risk level**							
	**Less than 10%**		**10-50%**		**More than 50%**		**All**	
	**n**	**%**	**n**	**%**	**n**	**%**	**n**	**%**
Less than 10%	183	85.5	31	14.5	0	0	214	5.3
10-50%	221	6.4	2992	87.2	219	6.4	3432	85.8
More than 50%	0	0	122	34.6	231	65.4	353	8.8

This table depicts the classification differences between the extended model (with pathology) to the model without pathology. Using a cut-off of less than 10% risk of 5 year all cause mortality. The extended model reclassified 31 of 214 (14.5%) to a higher risk group (between 10-50% risk). Using a cutoff of more than 50% the extended model moved 122 (34.6%) of 353 patients to a lower risk group. Of the 3432 patients classified to 10-50% risk the extended model moved 221 (6.4%) to the lower risk group and 219 (6.4%) to the higher risk group.

The multivariable models to predict prostate-cancer specific mortality are described in Tables 5 and 6 (Table 5- the model consisting of variable derived from administrative data only; Table 6- the model that included Gleason grade and cancer volume in addition to the variables in the administrative data model). Higher Gleason grade and cancer volume were important predictors of prostate cancer specific mortality. The accuracy of the models (i.e. c-statistic) to predict 5-year mortality were 0.76 (95% CI 0.74-0.78) and 0.85 (95% CI 0.83-0.87) for the admin-data only and the extended model (including pathology information), respectively. This corresponded to an incremental increase of 0.09 (95% CI 0.07-1.1) in the c-statistic.

**Table 5:** **Administrative data only model to predict prostate cancer specific mortality**

		**HR 95% CI**	**p**
Age		1.104 (1.08-1.13)	<0.0001
Year of cohort entry		0.815 (0.79-0.84)	<0.0001
Rural		1.29 (0.97- 1.97)	0.0747
Co morbidity ADGs	Low	Ref	
	Intermediate	1.26( 0.95-1.67)	0.106
	High	1.38 (1.04-1.82)	0.021
SES status	1	Ref	
	2	0.86 (0.62-1.2)	0.38
	3	1.003 (0.72-1.39)	0.98
	4	0.81 (0.56-1.16)	0.24
	5	0.93 (0.66-1.3)	0.68

Model c statistic to predict 5 year prostate cancer specific mortality: 0.76 (95% CI 0.74-0.78).

**Table 6:** **Extended model with pathology to predict prostate cancer specific mortality**

		**HR 95% CI**	**p**
Age		1.084 (1.06-1.106)	<0.0001
Year of cohort entry		0.806 (0.78-0.83)	<0.0001
Rural		1.31 (0.99-1.74)	0.054
Co morbidity ADGs	Low	Ref	
	Intermediate	1.33 (1.004-1.77)	0.047
	High	1.49 (1.1-1.97)	0.0043
SES status	1	Ref	
	2	0.846 (0.60-1.18)	0.32
	3	1.2 (0.87-1.68)	0.26
	4	0.85 (0.59-1.21)	0.36
	5	0.88 (0.63-1.25)	0.49
Gleason grade	Low	Ref	
	Intermediate	1.66 (1.14-2.4)	0.0076
	High	5.97 (4.2-8.47)	<0.0001
Volume of prostate cancer	Low (≤30%)	Ref	
	High ( >30%)	1.62 (1.23-2.33)	0.0012

Model c statistic to predict 5 year prostate cancer mortality: 0.85 (95% CI 0.83-0.87).

Using the NRI method, we first used the model based on administrative data only, to classify persons’ predicted probability of 5-year mortality into low (less the 10%), intermediate (10-50%) or high (more than 50%) risk. The risk category of predicted probability of 5-year prostate cancer specific mortality of 928 subjects (28%) in our cohort changed when the extended model was applied (Table 7). Among the 2981 subjects with low predicted probability of 5-year prostate cancer specific mortality (less than 10%), the extended model reclassified 378 (14.5%) to a higher risk group (Table 7). Among the 28 patients with a high predicted probability of 5-year prostate cancer specific mortality (more than 50%), the extended model moved 18 (64.3%) of to a lower risk group. Of the 990 patients classified to 10-50% risk the extended model moved 469 (47.4%) to the lower risk group and 58 (5.9%) to the higher risk group.

**Table 7:** **Net reclassification improvement**

**Basic model risk levels**	**Extended model risk level**							
	**Less than 10%**		**10-50%**		**More than 50%**		**All**	
	**n**	**%**	**n**	**%**	**n**	**%**	**n**	**%**
Less than 10%	2603	87.3	378	12.68	0	0	2981	74.54
10-50%	469	47.37	463	46.77	58	5.9	990	24.76
More than 50%	0	0	18	64.3	10	35.7	28	0.7

This table depicts the classification differences between the extended model (with pathology) to the model without pathology. Using a cut-off of less than 10% risk of 5 year prostate-cancer specific mortality. The extended model reclassified 378 of 2981 (12.68%) to a higher risk group (between 10-50% risk). Using a cutoff of more than 50% the extended model moved 18 (64.3.6%) of 28 patients to a lower risk group. Of the 990 patients classified to 10-50% risk the extended model moved 469 (47.37%) to the lower risk group and 58 (5.9%) to the higher risk group.

Using data from 4001 elderly male diabetic patients who subsequently developed prostate cancer, we demonstrated that pathology data obtained by chart abstraction improved the accuracy in predicting all-cause and prostate-cancer specific mortality. The benefit in predicting all-cause mortality was modest, evident by only a 0.04 difference in the c-statistic, and by the fact that the extended model (with Gleason grade and cancer volume) changed the risk category of predicted probability of survival for 14.8% of the men in our cohort. In contrast to this, pathology data modified the accuracy in predicting prostate cancer specific mortality considerably. The extended model demonstrated a c-statistic of 0.85 (95% CI 0.83-0.87) compared to 0.76 (95% CI 0.74-0.78) when only administrative data was used. Furthermore, the extended model changed the risk category of predicted probability of 5 years survival for 28% of the men in our cohort.

Most predictive models in prostate cancer rely on clinical data such as PSA, Gleason grade, cancer volume and stage [32-34]. Detailed clinical information is often missing in large administrative data, and therefore these models have limited use among policy makers and health service researchers. Deriving missing data from administrative database can be accomplished in several methods. One could abstract a random sample of medical records and use that abstraction to develop an algorithm to infer the data [35]. However this rarely applies to pathology. There is no unified clinical pathway that may identify Gleason grade or volume of tumor. For example two patients with a similar Gleason grade and stage may receive different treatments, and vice versa patients with different pathology may receive similar treatment. Though less efficient, we believe that chart review is the most reliable method for pathology data gathering.

In this study we demonstrated that the value of chart review and detailed pathology data depends on the research question and the accuracy threshold that is acceptable. Changes in NRI risk categories correspond to change in sensitivity at the higher threshold plus change in sensitivity at the lower threshold (personal communication Michael Pencina). For example, if 10% accuracy is needed then chart abstraction is required regardless of outcome. However, in a study were 20% accuracy is acceptable, we demonstrated that chart review is needed only for the outcome of prostate cancer mortality; and not for all-cause mortality.

In our study, we reviewed over 5000 pathology reports- if one assumes a trained chart abstractor (that costs approximately $25 an hour) can review a report in 10 minutes, the total time dedicated to chart review was 840 hours. The total cost associated with this endeavor was $21,000. This may not be feasible for a larger cohort.

Two key aspects of prediction model performance are discrimination and calibration [36]. Discrimination refers to the ability of a prediction model to distinguish between patients. A typical measure of discrimination is the c-statistic; the c-statistic provides the probability that, for a randomly selected pair of subjects, the model gives a higher probability to the subject who had the event, or who had the shorter survival time. However, one limitation of the c-statistic is that a strong risk predictor may have limited impact [31,36]. Furthermore, the c-statistic is difficult to interpret clinically. Therefore, in our study we used NRI to assess the difference in calibration between the models. Calibration measures whether predicted probabilities agree with observed proportions. Reclassification can directly compare the clinical impact of two models by determining how many individuals would be reclassified into clinically relevant risk strata. Adding Gleason grade and cancer volume to the prediction model for all-cause mortality moved approximately 15% of subjects- however adding the same variables moved approximately 30% of subjects in the model predicting prostate-cancer specific mortality. There are many other tests that may be used to measure predictive accuracy such as calibration plots, decision curves, and integrated discrimination improvement [37-40]. Since the objective of our study was to establish the importance of pathological data and not form the ideal prediction model we did not utilize all these measures.

In our NRI analysis we used several cut-offs to assess reclassification. We considered the 5-year probability of mortality as low (less the 10%), intermediate (10-50%) or high (more than 50%) risk. Although risk is a continuum, clinicians ultimately have to make binary choices, such as whether or not to treat a subject. This entails consideration of how high a risk is “high enough” to necessitate action. A risk cut-point depends on the relationship between the harms of an event and the harms of needless treatment. We therefore set our cut-points to be clinically relevant. Although the intermediate risk category range is rather large (10-50% risk), these patients are often grouped together and treatment decisions are made at the extremes. Since we aim to assess the utility of adding clinical variables to a prediction model, we believe that these categories are sufficient.

There are several limitations to our study. First, our population was older, diabetic, and had worse Gleason grade distribution than the general population of PC patients, thus generalizability to a contemporary PC cohort is tempered. It is possible that among a younger cohort pathology may have a greater impact on both all-cause mortality and prostate cancer-specific mortality. However, nearly three quarters of men with prostate cancer are aged 65 and older at the time of diagnosis and most of these patients have other comorbidities such as hypertension, ischemic heart disease, diabetes, etc. [9,13,15] Restricting our cohort to incident diabetics who subsequently develop prostate cancer served to create a more homogeneous cohort and minimize the possibility for misclassification of comorbidities a common problem with administrative data [41,42]. Second, we did not include treatment (such as radiation or surgery) in our predictive models. Since these variables are post baseline (i.e. they occur after the diagnosis) they need to be modeled as time-dependent covariants. If they are not modeled appropriately that introduces immortal bias, since in order to receive a treatment one obviously needs to survive until that time. Since there is no known way of calculating the c-statistic or NRI for Cox proportional models with time dependent covariates we have decided not to include them in our model. Furthermore, since treatment would have been included in both the administrative data and the extended models, their exclusion should not have changed our results. Finally, we lack data on severity of diabetes, body mass index, and prostate cancer stage even in the model including pathology. Further studies are needed to address the impact of adding these variables.

## Conclusions

Administrative data are relatively inexpensive to obtain. In contrast, abstracting clinical data from patients’ medical records is expensive, time consuming and labor intensive. The current study demonstrates that the additional cost associated with the use of detailed clinical data may not be justified for an outcome of all-cause mortality, but is more important to study prostate-cancer specific mortality. This information is useful to inform administrative health researchers and policy-makers about the proper allocation of funding resources.

## References

[1] Rochon PA, Tu JV, Anderson GM, Gurwitz JH, Clark JP, Lau P, Szalai JP, Sykora K, Naylor CD (2000). Rate of heart failure and 1-year survival for older people receiving low-dose beta-blocker therapy after myocardial infarction. Lancet.

[2] Soumerai SB, McLauglin TJ, Spiegelman D, Hertzmark E, Thibault G, Goldman L (1997). Adverse outcomes of underuse of beta-blockers in elderly survivors of acute myocardial infarction. JAMA.

[3] Mamdani MM, Rochon PA, Juurlink DN, Kopp A, Anderson GM, Naglie G, Austin PC, Laupacis A (2002). Observational study of upper gastrointestinal haemorrhage in elderly patients given selective cyclo-oxygenase- inhibitors or conventional non-steroidal anti-inflammatory drugs. BMJ.

[4] McClellan M, McNeil BJ, Newhouse JP (1994). Does more intensive treatment of acute myocardial infarction in the elderly reduce mortality?. JAMA.

[5] Valentini V, van Stiphout RG, Lammering G, Gambacorta MA, Barba MC, Bebenek M, Bonnetain F, Bosset JF, Bujko K, Cionini L, Gerard JP, Rödel C, Sainato A, Sauer R, Minsky BD, Collette L, Lambin P (2011). Nomograms for predicting local recurrence, distant metastases, and overall survival for patients with locally advanced rectal cancer on the basis of European randomized clinical trials. J Clin Oncol.

[6] Kattan MW, Karpeh MS, Mazumdar M, Brennan MF (2003). Postoperative nomogram for disease-specific survival after an R0 resection for gastric carcinoma. J Clin Oncol.

[7] Ferrone CR, Kattan MW, Tomlinson JS, Thayer SP, Brennan MF, Warshaw AL (2005). Validation of a postresection pancreatic adenocarcinoma nomogram for disease-specific survival. J Clin Oncol.

[8] Carver JR, Shapiro CL, Ng A, Jacobs L, Schwartz C, Virgo KS, Hagerty KL, Somerfield MR, Vaughn DJ, ASCO Cancer Survivorship Expert Panel (2007). American Society of Clinical Oncology clinical evidence review on the ongoing care of adult cancer survivors: cardiac and pulmonary late effects. J Clin Oncol.

[9] Seigel R, Ward E, Brawley O, Jemal A (2011). Cancer Statistics, 2011. CA Cancer J Clin.

[10] GLOBOCAN: *Prostate Cancer Incidence and Mortality Worldwide in 2008.* 2008.

[11] Wong YN, Mitra N, Hudes G, Localio R, Schwartz JS, Wan F, Montagnet C, Armstrong K (2006). Survival associated with treatment vs observation of localized prostate cancer in elderly men. JAMA.

[12] Kim MM, Hoffman KE, Levy LB, Frank SJ, Pugh TJ, Choi S, Nguyen QN, McGuire SE, Lee AK, Kuban DA (2012). Prostate cancer-specific mortality after definitive radiation therapy: Who dies of disease?. Eur J Cancer.

[13] Abdollah F, Sun M, Schmitges J, Tian Z, Jeldres C, Briganti A, Shariat SF, Perrotte P, Montorsi F, Karakiewicz PI (2011). Cancer-specific and other-cause mortality after radical prostatectomy versus observation in patients with prostate cancer: competing-risks analysis of a large North American population-based cohort. Eur Urol.

[14] Shetti MB, Merrick GS, Butler WM, Galbreath R, Torlone A, Lief JH, Adamovich E, Wallner KE (2011). The Impact of Diabetes Mellitus on Survival in Men With Clinically Localized Prostate Cancer Treated With Permanent Interstitial Brachytherapy. Am J Clin Oncol.

[15] Chamie K, Daskivich TJ, Kwan L, Labo J, Dash A, Greenfield S, Litwin MS (2012). Comorbidities, Treatment and Ensuing Survival in Men with Prostate Cancer. J Gen Intern Med.

[16] Margel D, Urbach D, Lipscombe LL, Bell CM, Kulkarni G, Austin PC, Fleshner N (2013). Metformin and prostate cancer and all-cause mortality among diabetic men. JCO.

[17] Robles SC, Marrett LD, Clarke EA, Risch HA (1988). An application of capture recapture methods to the estimation of completeness of cancer registration. J Clin Epidemiol.

[18] Williams JI, Young W, Goel V, Williams JI, Anderson GM, Blacksterin-Hirsch P, Fooks C, Naylor CD (1996). A summary of studies on the quality of health care administrative databases in Canada. Patterns of Health Care in Ontario. The ICES Practice Atlas.

[19] Juurlink D, Preyra C, Croxford R (2006). Canadian Institute for Health Information Discharge Abstract Database: a validation study.

[20] Hux JE, Ivis F, Flintoft V, Bica A (2002). Diabetes in Ontario: determination of prevalence and incidence using a validated administrative data algorithm. Diabetes Care.

[21] Brenner DR, Tammemägi MC, Bull SB, Pinnaduwaje D, Andrulis IL (2009). Using cancer registry data: agreement in cause-of-death data between the Ontario Cancer Registry and a longitudinal study of breast cancer patients. Chronic Dis Can.

[22] Hall S, Schulze K, Groome P, Mackillop W, Holowaty E (2006). Using cancer registry data for survival studies: the example of the Ontario Cancer Registry. J Clin Epidemiol.

[23] Schouten LJ, Jager JJ, van den Brandt PA (1993). Quality of cancer registry data: a comparison of data provided by clinicians with those of registration personnel. Br J Cancer.

[24] Austin PC, Walraven C (2011). The mortality risk score and the ADG score: two points-based scoring systems for the Johns Hopkins aggregated diagnosis groups to predict mortality in a general adult population cohort in Ontario, Canada. Med Care.

[25] Austin PC, van Walraven C, Wodchis WP, Newman A, Anderson GM (2011). Using the Johns Hopkins Aggregated Diagnosis Groups (ADGs) to predict mortality in a general adult population cohort in Ontario, Canada. Med Care.

[26] Pencia MJ, D’Agostino RB, D’Agostino RB (2008). Evaluating the added predictive ability of a new marker. From area under the ROC curve to reclassification and beyond. Stat Med.

[27] Harrell FE, Kee KL, Mark DB (1996). Multivariable prognostic models: issues in developing models, evaluating assumptions and adequacy, and measuring and reducing errors. Stat Med.

[28] Pencina MJ, D’Agostino RB (2004). Overall C as a measure of discrimination in survival analysis: model specific population value and confidence interval estimation. Stat Med.

[29] Steyerberg EW, Pencina MJ (2010). Reclassification calculations for persons with incomplete follow-up. Ann Intern Med.

[30] Kettermann AE, Ferrucci L, Trock BJ, Metter EJ, Loeb S, Carter HB (2010). Interpretation of the prostate-specific antigen history in assessing life-threatening prostate cancer. BJU Int.

[31] Chambless LE, Cummiskey CP, Cui G (2011). Several methods to assess improvement in risk prediction models: extension to survival analysis. Stat Med.

[32] Shariat SF, Karakiewicz PI, Roehrborn CG, Kattan MW (2008). An updated catalog of prostate cancer predictive tools. Cancer.

[33] Eggener SE, Scardino PT, Walsh PC, Han M, Partin AW, Trock BJ, Feng Z, Wood DP, Eastham JA, Yossepowitch O, Rabah DM, Kattan MW, Yu C, Klein EA, Stephenson AJ (2011). Predicting 15-year prostate cancer specific mortality after radical prostatectomy. J Urol.

[34] Cooperberg MR, Hilton JF, Carroll PR (2011). The CAPRA-S score: A straightforward tool for improved prediction of outcomes after radical prostatectomy. Cancer.

[35] Seeger JD, West WA, Fife D, Noel GJ, Johnson LN, Walker AM (2006). Achilles tendon rupture and its association with fluoroquinolone antibiotics and other potential risk factors in a managed care population. Pharmacoepidemiol Drug Saf.

[36] Vickers AJ: **Prediction models in cancer care.** *CA Cancer J Clin.* 2011, **23:** doi:10.3322/caac.20118. [Epub ahead of print] 10.3322/caac.20118 PMC3189416 21732332

[37] Vickers AJ, Elkin EB (2006). Decision curve analysis: a novel method for evaluating prediction models. Med Decis Making.

[38] Cook NR, Ridker PM (2009). Advances in measuring the effect of individual predictors of cardiovascular risk: the role of reclassification measures. Ann Intern Med.

[39] Steyerberg EW, Vickers AJ, Cook NR, Gerds T, Gonen M, Obuchowski N, Pencina MJ, Kattan MW, Gerds T, Gonen M, Obuchowski N, Pencina MJ, Kattan MW (2010). Assessing the performance of prediction models: a framework for traditional and novel measures. Epidemiology.

[40] Vickers AJ, Cronin AM (2010). Everything you always wanted to know about evaluating prediction models (but were too afraid to ask). Urology.

[41] Ladouceur M, Rahme E, Pineau CA, Joseph L (2007). Robustness of prevalence estimates derived from misclassified data from administrative databases. Biometrics.

[42] Benchimol EI, Manuel DG, To T, Griffiths AM, Rabeneck L, Guttmann A (2011). Development and use of reporting guidelines for assessing the quality of validation studies of health administrative data. J Clin Epidemiol.

